# Role of the Cervical Anterior Spinal Artery in the Endovascular Treatment of Vascular Diseases: Bystander, Accomplice, Victim, or Friend?

**DOI:** 10.3389/fneur.2021.761006

**Published:** 2021-10-26

**Authors:** Kun Zhang, Chao Li, Kun Hou, Jinlu Yu

**Affiliations:** ^1^Department of Cerebrovascular Disease, Henan Provincial People's Hospital, Zhengzhou University, Zhengzhou, China; ^2^Department of Neurology, First Hospital of Jilin University, Changchun, China; ^3^Department of Neurosurgery, First Hospital of Jilin University, Changchun, China

**Keywords:** cervical anterior spinal artery, vascular disease, endovascular treatment, arteriovenous malformation, arteriovenous fistula

## Abstract

The cervical anterior spinal artery (ASA) is a very important artery arising from the intracranial vertebral artery (VA). It can play different roles in endovascular treatment (EVT) of spinal vascular diseases. The current understanding of these roles is incomplete; therefore, we performed this review. We found that cervical ASA can be involved in many spinal vascular diseases, such as arteriovenous fistula (AVF), arteriovenous malformation (AVM), and aneurysm, and can serve as a collateral channel in proximal VA occlusion. In AVF and AVM, when the cervical ASA is involved, it often plays the role of an accomplice or victim because it acts as the feeder or as a bystander that does not provide blood flow to the AVF and AVM. In cervical ASA aneurysm, the ASA is a victim. During EVT of VA aneurysms or stenoses, the cervical ASA ostia can be covered or occluded, resulting in ASA ischemia. In this situation, the ASA is a victim. In VA occlusion or the subclavian steal phenomenon, the cervical ASA can serve as a collateral channel to provide blood flow to the posterior circulation. In this case, the ASA plays the role of a friend. According to the role of the cervical ASA in spinal vascular diseases, EVT should be determined “case by case.” Most importantly, when EVT is performed to treat these diseases, the cervical ASA axis must be preserved. Therefore, understanding the role of the cervical ASA in spinal vascular diseases is crucial.

## Introduction

The cervical anterior spinal artery (ASA) arises from the intracranial vertebral artery (VA) and provides blood to the anterior two-thirds of the spinal cord (1). It may be involved in many cervical spinal vascular diseases, such as arteriovenous fistula (AVF), arteriovenous malformation (AVM), and aneurysm (2–4). In addition, in VA occlusion or the subclavian steal phenomenon, the cervical ASA can serve as a collateral channel (5).

Currently, endovascular treatment (EVT) has become an effective method for cervical spinal vascular diseases (6). However, the EVT can damage the cervical ASA. For instance, during flow diversion (FD) deployment, VA stent angioplasty, or balloon angioplasty, the cervical ASA ostia can be covered or occluded (7). Therefore, the role of the cervical ASA is very complex. It can be an accomplice, victim, bystander, or friend.

According to the role of the cervical ASA in spinal vascular diseases, EVT should be determined “case by case.” It is of most importance that the cervical ASA axis be preserved (8). The current understanding of the role of the cervical ASA in EVT of spinal vascular diseases is insufficient. Therefore, we performed this important review.

## Cervical ASA Anatomy

The typical ASA originates as a common trunk from paired VAs, and its origin is 5–17 mm proximal to the vertebrobasilar junction, angiographically presenting with a characteristic midline hairpin (5, 9, 10). High anatomical variability of the ASA origin exists, and it often has either a predominance of one ramus over the other or a sole unilateral ramus of origin (11, 12). The Santos-Franco et al. study reported a less typical bilateral origin of the ASA (13).

The ASA is not a single artery but a series of anastomotic vascular loops. In the cervical region, the ASA is continuous, unlike that in the thoracic region (12, 14, 15). The cervical ASA requires segmental radiculomedullary arteries from the VA (C1–C6), the ascending cervical artery (C3–C4), and the deep cervical artery (C3–C7) (10, 16, 17). In the craniocervical junction, the ascending pharyngeal artery (C2–C4) and the occipital artery (C1–C2) can be involved in the blood supply to the ASA (18, 19). In addition, the supreme intercostal artery can be involved as a feeder to the ASA in the cervical region (C7) (20).

**Figure 1:**
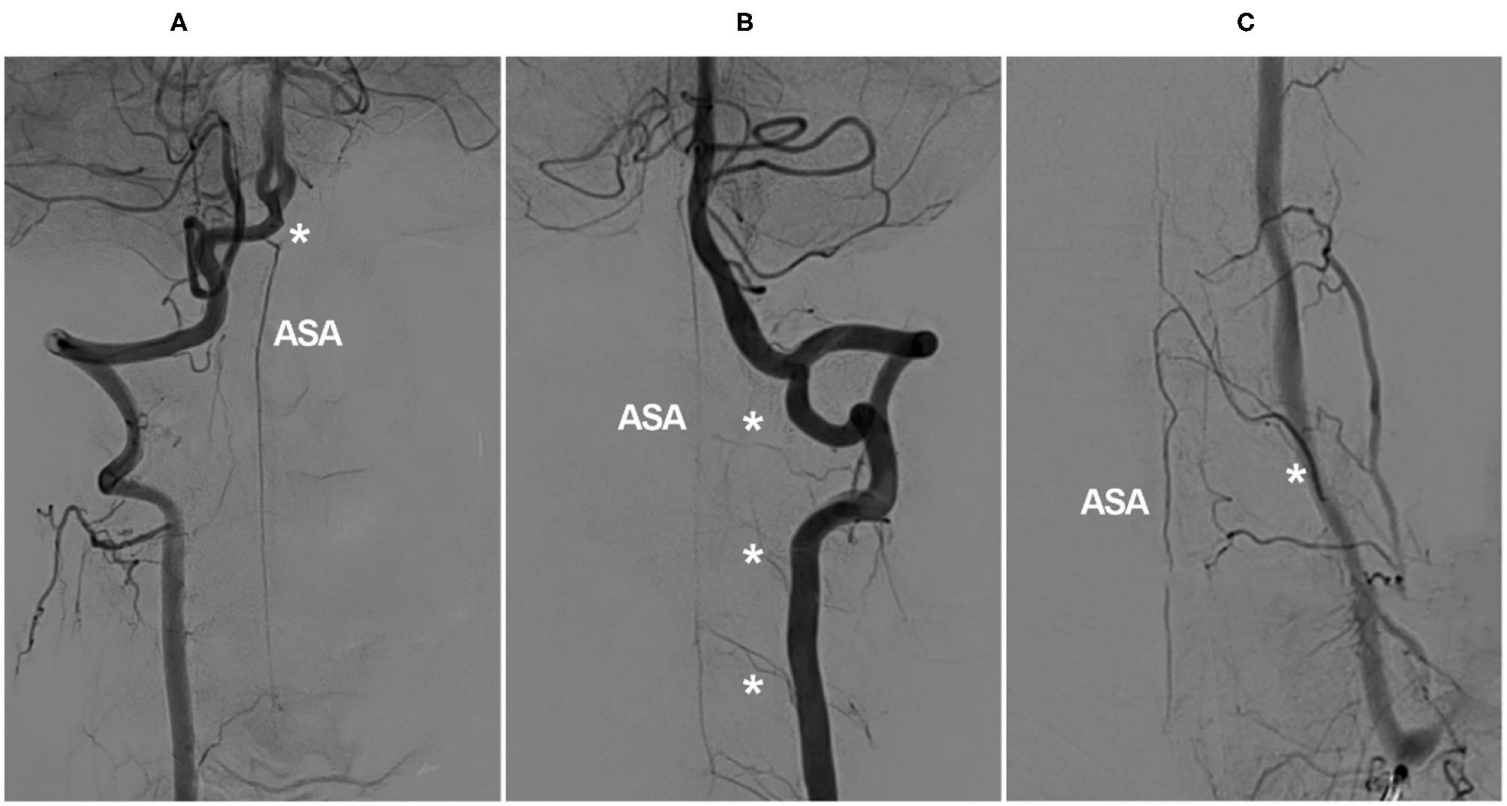
Angiographic cervical ASA anatomy. **(A)** VA angiogram showing a long-distance ASA arising from intracranial VA termination (asterisk). **(B)** VA angiogram showing that the ASA is relayed by the continuous segmental radiculomedullary arteries (asterisks). **(C)** VA angiogram showing the artery of cervical enlargement (asterisk). ASA, anterior spinal artery; VA, vertebral artery.

In the cervical region, the radiculomedullary artery from C4–C7 is usually the most predominant and is called the artery of cervical enlargement (16, 21). In addition to the VA, the artery of cervical enlargement can arise as a segmental branch of the ascending or deep cervical arteries (22–24). The angiographic anatomy of the cervical ASA is shown in Figure 1.

## Cervical Spinal AVFs

Cervical spinal AVFs vary and can be divided into dural AVFs (DAVFs), radicular AVFs, epidural AVFs, and perimedullary AVFs (PAVFs); they are located on the inner or outer surface of the dura, on the spinal nerves, or on the spinal cord (6, 25–28). In cervical AVFs, the ASA can be an active accomplice. In the Hiramatsu et al., study, half of high cervical AVFs were fed by the ASA (6).

### Cervical DAVFs

Spinal DAVFs are located near or within the dura of the nerve sleeve, connecting radiculomeningeal arteries with the radicular vein that drains into the perimedullary vein (29). Cervical DAVFs occur in <6% of spinal DAVFs (30–33). In most cervical DAVFs, the ASA acts as a bystander (34–36). Rarely, the ASA can be an accomplice. In the report of Adrianto et al., 2.4% of cervical DAVFs had a concomitant origin of the ASA with the feeder (37).

Currently, EVT is feasible (31, 32). However, when cervical DAVFs originate from the radicular branch that supplies both the fistula and ASA, the EVT must be chosen carefully (31). During EVT, liquid embolic materials can be chosen, and they should penetrate the vein beyond the fistula without disturbing the ASA. Therefore, the microcatheter should be in a wedged position to ensure no contrast reflux into the ASA (36). In EVTs for other AVFs and AVMs, the microcatheter should be in the wedged position.

For cervical DAVFs, the liquid material N-butyl-2-cyanoacrylate (NBCA) and Onyx (Medtronic, Irvine, California, USA) can be used, and NBCA with a lipiodol mixture at a 20–30% concentration is preferred (31). Onyx is limited by the difficulty in achieving venous penetration; at this time, using a balloon-occlusion catheter to assist Onyx casting is helpful, which can promote Onyx penetration (38).

### Radicular AVFs

Radicular AVFs are located on the intradural nerve root and are fed by radicular and/or radiculomeningeal arteries that drain into the radicular vein (39, 40). Cervical radicular AVFs have an angioarchitecture similar to that of spinal DAVFs. However, they are different. First, the radicular AVF site is on the nerve root, and the C1 or C2 level is the preferred location (39, 40). Second, in radicular AVFs, the ASA often joins the same radicular artery (6, 40). Third, in radicular AVFs, a long-distance perimedullary draining vein is uncommon (6, 39, 40). Because radicular AVFs often have an ASA blood supply, EVT has a higher risk.

### Cervical Epidural AVFs

**Figure 2:**
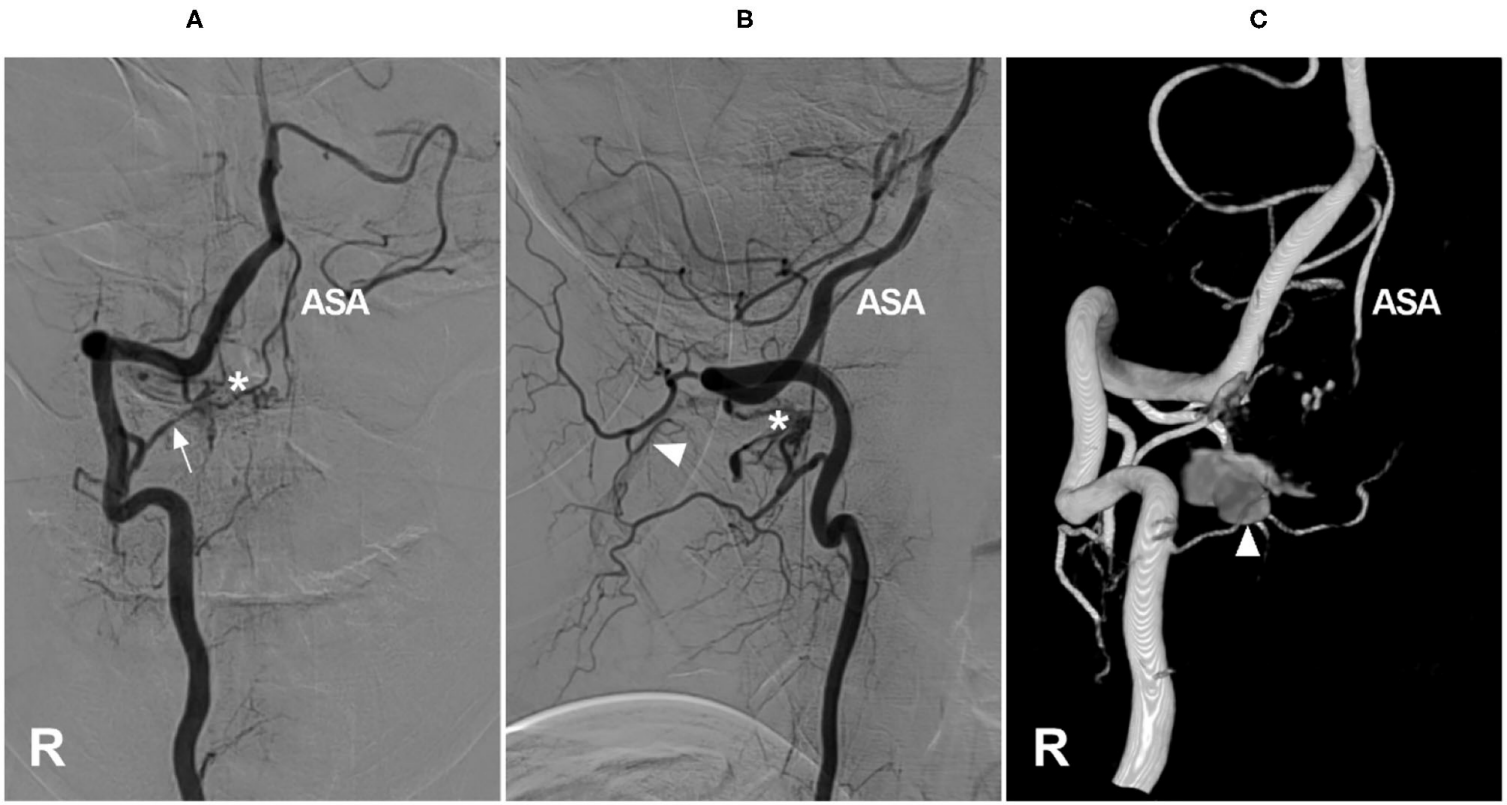
Cervical epidural arteriovenous fistula. **(A–C)** Right VA anteroposterior view **(A)**, lateral view **(B)**, and three-dimensional **(C)** angiograms showed an epidural arteriovenous fistula (asterisk), the arrow shows the feeding artery and the ASA were involved, with draining to the suboccipital venous plexus (triangle). ASA, anterior spinal artery; R, right; VA, vertebral artery.

Spinal epidural AVFs feature a dilated epidural venous pouch that is supplied by paraspinal or paravertebral arteries and drains into epidural plexuses (41–43). Cervical epidural AVFs are not uncommon (Figure 2). In the report of Asai et al., 30.8% of epidural AVFs were located in the cervical region and often in the lateral spinal canal, even with bone involvement (44–46). They are divided into types A and B: type A has a small venous pouch with intradural venous drainage, often with congestive myelopathy, and type B has a large venous lake without intradural drainage, often with compressive myelopathy (47–50).

In cervical epidural AVFs, especially in high locations, spinal pial arteries often have a common origin with the feeder; therefore, the ASA can be involved as an accomplice (50, 51). In the Hiramatsu et al., study, 57% of high cervical epidural AVFs were fed by the ASA (6).

EVT is appropriate for cervical epidural AVFs. Transarterial EVT is mostly applied, and liquid embolic material must be avoided in the ASA (48, 52, 53). Sometimes, transarterial EVT of high/middle cervical epidural AVFs is difficult, as these AVFs are usually fed by small and short VA branches (48, 54). If there is a fistulous connection, transvenous EVT is a good choice, and the use of a proximal balloon to control high blood flow followed by coiling or liquid embolic material embolization is helpful (51, 55).

### Cervical PAVFs

PAVFs result from direct communication between feeding arteries and enlarged draining veins without the intervening nidus; they are intradural but extramedullary, and in the cervical region, they are usually located on the anterior or lateral surface of the spinal cord (56). The shunting points can be single or multiple (57). Cervical PAVFs are not uncommon; in the Mizutani et al., study, PAVFs in the cervical region accounted for 22.4% of all PAVFs (2). In the study of Mizutani et al., of pediatric cases, the rate was 11.8% (2).

The ASA is an active accomplice in cervical PAVFs. In the Endo et al. study of 22 cervical PAVFs, the ASA contributed to shunts in 72.7% of patients (58). Cervical PAVFs can be divided into three types. Type A PAVFs are small, single-vessel fistulas supplied by a single ASA that mostly occur in adult patients. Types B and C PAVFs are giant, multiple-vessel fistulas supplied by the ASA and posterolateral spinal arteries, with high-flow, enlarged, and tortuous draining veins that occur more often in children (59, 60).

**Figure 3:**
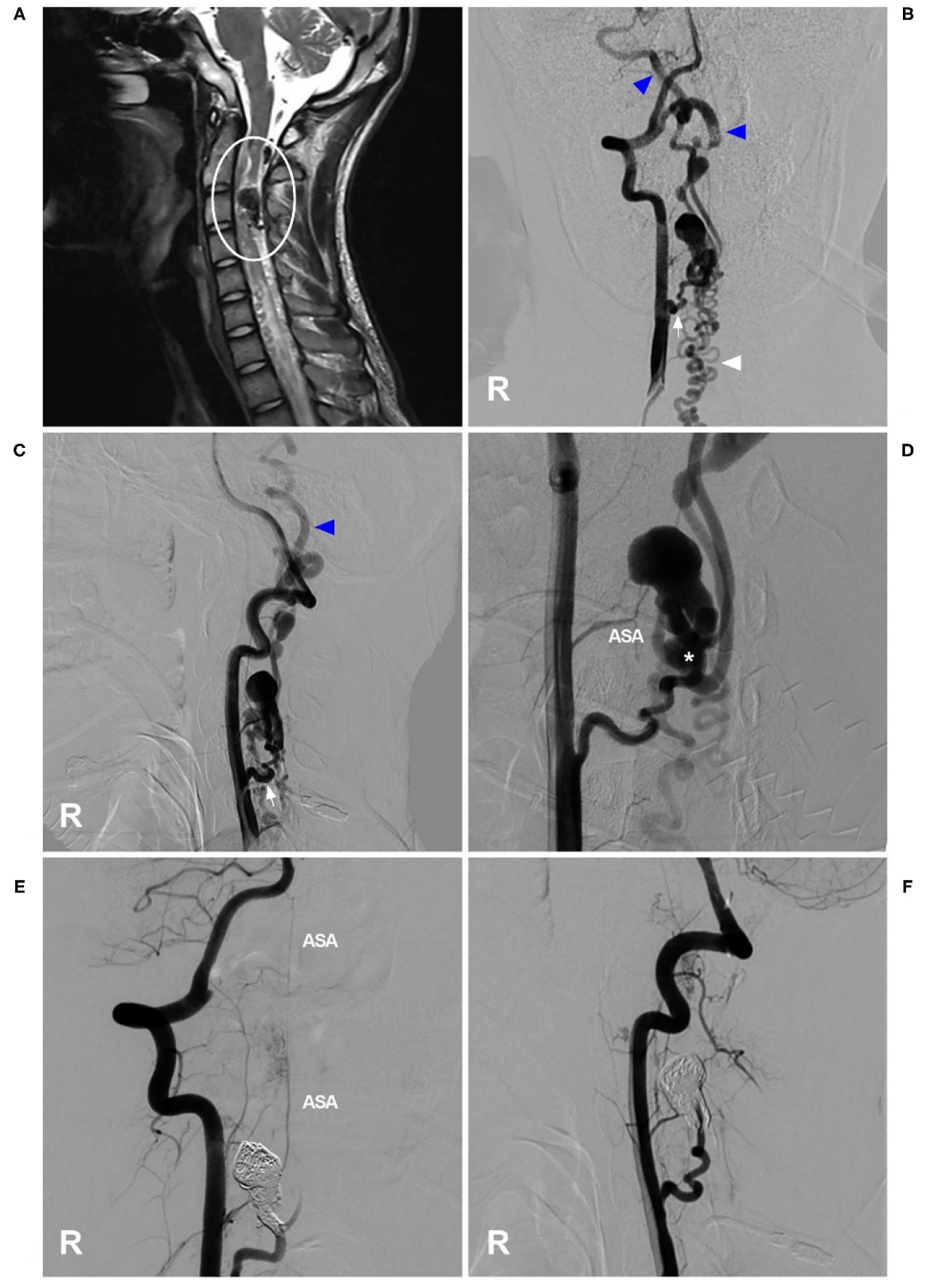
EVT of a cervical perimedullary arteriovenous fistula. **(A)** MRI showed a PAVF near the C3 level (circle). **(B,C)** Right VA anteroposterior view **(B)** and lateral view **(C)** angiograms showed that the PAVF was mainly supplied by a dilated radiculomeningeal artery (arrow), with draining to the cranium (blue triangles) and lower neck (white triangle), and that the draining veins were dilated. **(D)** An angiogram showed the shunt point of the PAVF (asterisk). The ASA could be seen as a bystander. **(E,F)** Right VA anteroposterior view **(E)** and lateral view **(F)** postoperative angiograms showed that the PAVF was embolized by coiling. The ASA remained intact as a bystander. ASA, anterior spinal artery; EVT, endovascular treatment; MRI, magnetic resonance imaging; PAVF, perimedullary arteriovenous fistula; R, right; VA, vertebral artery.

Cervical PAVFs can be managed with EVT, and the key aim is ASA preservation as long as possible during fistula obliteration (58, 61, 62). Not all PAVFs are appropriate for EVT. For Type A PAVFs, EVT is difficult because ASA catheterization is problematic; for Types B and C PAVFs, transarterial EVT via the ASA may be a safe and effective choice (57, 63). Certainly, if the ASA is a bystander in PAVFs, EVT is easy (Figure 3). In addition, the transvenous path can be used (58). Coils and NBCA are preferred for cervical PAVFs because of the short course of the ASA extending from the VA (64, 65).

## Cervical Intramedullary Glomus AVM

Spinal glomus AVM is an intramedullary arteriovenous shunt with an intervening nidus (60, 66). A cervical location accounts for ~ 30% of intramedullary glomus AVMs with multiple feeding vessels arising from the ASA and posterolateral spinal arteries (67–69). The vast majority of intramedullary glomus AVMs are diffuse, and they are comparatively smaller than juvenile-type AVMs (68). The ASA is often an active accomplice. In the Mizutani et al., study including 69 glomus intramedullary AVMs, 100% of the ASAs were involved (2).

**Figure 4:**
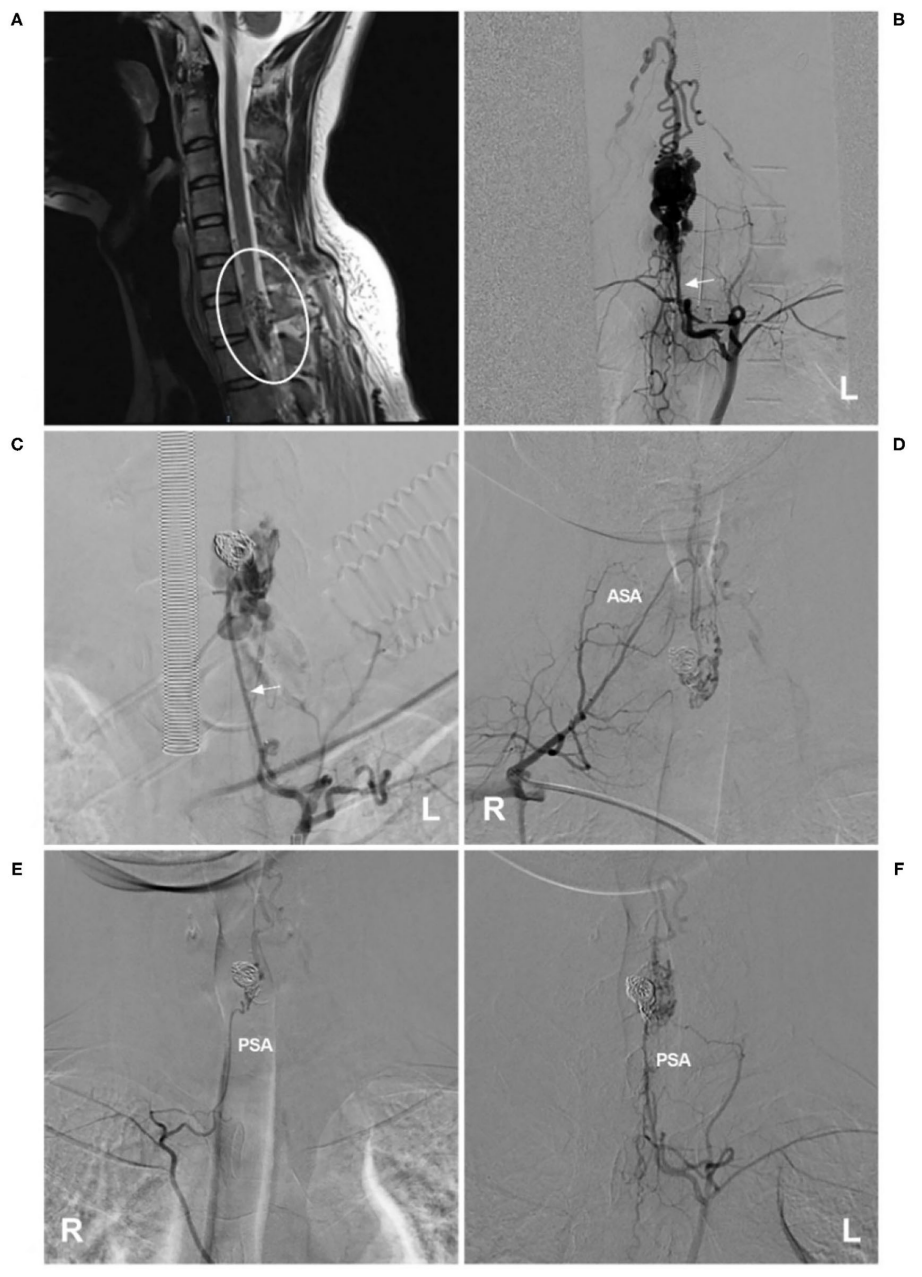
EVT of a cervical intramedullary glomus arteriovenous malformation. **(A)** MRI showed an intramedullary glomus AVM near the C7 level (circle). **(B)** Left angiogram of the supreme intercostal artery showed that the AVM was mainly supplied by the dilated radiculomeningeal artery (arrow). **(C)** The aneurysm in the AVM was coiled first *via* the radiculomeningeal artery (arrow), and then the NBCA was cast. **(D–F)** Six-month follow-up angiograms showed that the AVM had decreased. Radiotherapy was recommended. **(D)** shows an angiogram via the right costocervical trunk revealing that the ASA was involved as the feeder. **(E,F)** show angiograms via the left and right supreme intercostal arteries, revealing that the PSA was the feeder. AVM, arteriovenous malformation; ASA, anterior spinal artery; EVT, endovascular treatment; L, left; MRI, magnetic resonance imaging; NBCA, N-butyl cyanoacrylate; PSA, posterior spinal artery; R, right.

Currently, EVT is a good option for cervical glomus AVMs (70). For ideal EVT, only the nidus is eliminated, while the ASA axis is maintained (71). If the ASA is not chosen as the pathway to perform EVT, then EVT is easy (Figure 4). If EVT is performed via the ASA, the embolic agent used is NBCA or Onyx, provided that the microcatheter tip can be placed within the nidus (72–74). If the microcatheter tip can be placed close to the nidus but beyond the angiographically visible normal ASA, NBCA is still a good choice (75).

However, the goal of complete EVT is difficult because the main feeding vessel of the cervical glomus AVM, a perforating artery from the ASA, also serves as a feeding vessel to the spinal cord (76). EVT can be accompanied by inadvertent proximal reflux of the embolic material (72, 77). Therefore, many glomus AVMs cannot be cured by EVT; more often, EVT is adopted extensively as a palliative treatment (78).

## Cervical ASA Aneurysm

ASA aneurysms are rare and are mainly located in the upper cervical segment (4, 79–82). They are typically dissections and can be divided into isolated and flow-related types (83, 84). The flow-related type is common. In the Madhugiri et al., study, 20.8% of spinal aneurysms were associated with AVM, and 7.8% of aneurysms were isolated (80). Flow-related aneurysms are found in 29% of glomus AVMs and 10% of spinal PAVFs (85, 86). Even cervical epidural AVFs can be associated with ASA aneurysms (50).

EVT for isolated ASA aneurysms remains difficult because the catheter system must be constructed via the small-caliber ASA (3). For flow-related aneurysms of the ASA, because the ASA is often dilated, EVT may be feasible (87). When the aneurysm is located at the bifurcation between a large artery supplying the AVM and the ASA, the aneurysm may be suited for coiling (88). If liquid embolic material is used, the ASA axis needs to be preserved, and only superselective embolization of the branches harboring the aneurysm can be allowed (87, 89).

In addition, conservative management is a reasonable option for ASA aneurysms (90, 91). Even flow-related ASA aneurysms can be managed conservatively. In an Ichiro et al. report, a ruptured aneurysm of the ASA regressed after feeding to an AVF after EVT eliminated the AVF and reduced the hemodynamic stress on the aneurysm (50). The reduction of hemodynamic stress is effective, and even ruptured ASA aneurysms can be cured by hemodynamic remodeling with FD placed in the ipsilateral VA (92).

## Cervical ASA Ischemia from ASA Occlusion or Coverage by EVT

During EVT, the ASA ostia can be occluded or covered, resulting in medullary ischemia. The procedures include conventional stent-assisted coiling, VA trapping, FD deployment, VA stent angioplasty, etc. In such cases, the ASA is a victim.

### Stent-Assisted Embolization of VA Aneurysms and VA Trapping

According to Wang et al., VA aneurysms can be classified into three types: type I aneurysms, located distal to the posterior inferior cerebellar artery (PICA); type II aneurysms, located at the PICA origin; and type III aneurysms, located proximal to the PICA (93). In theory, EVT for type III aneurysms can result in occlusion of the ASA ostia. However, multiple segmental arterial supplies of the cervical spinal cord make the ASA relatively resistant to ischemia (94–96).

**Figure 5:**
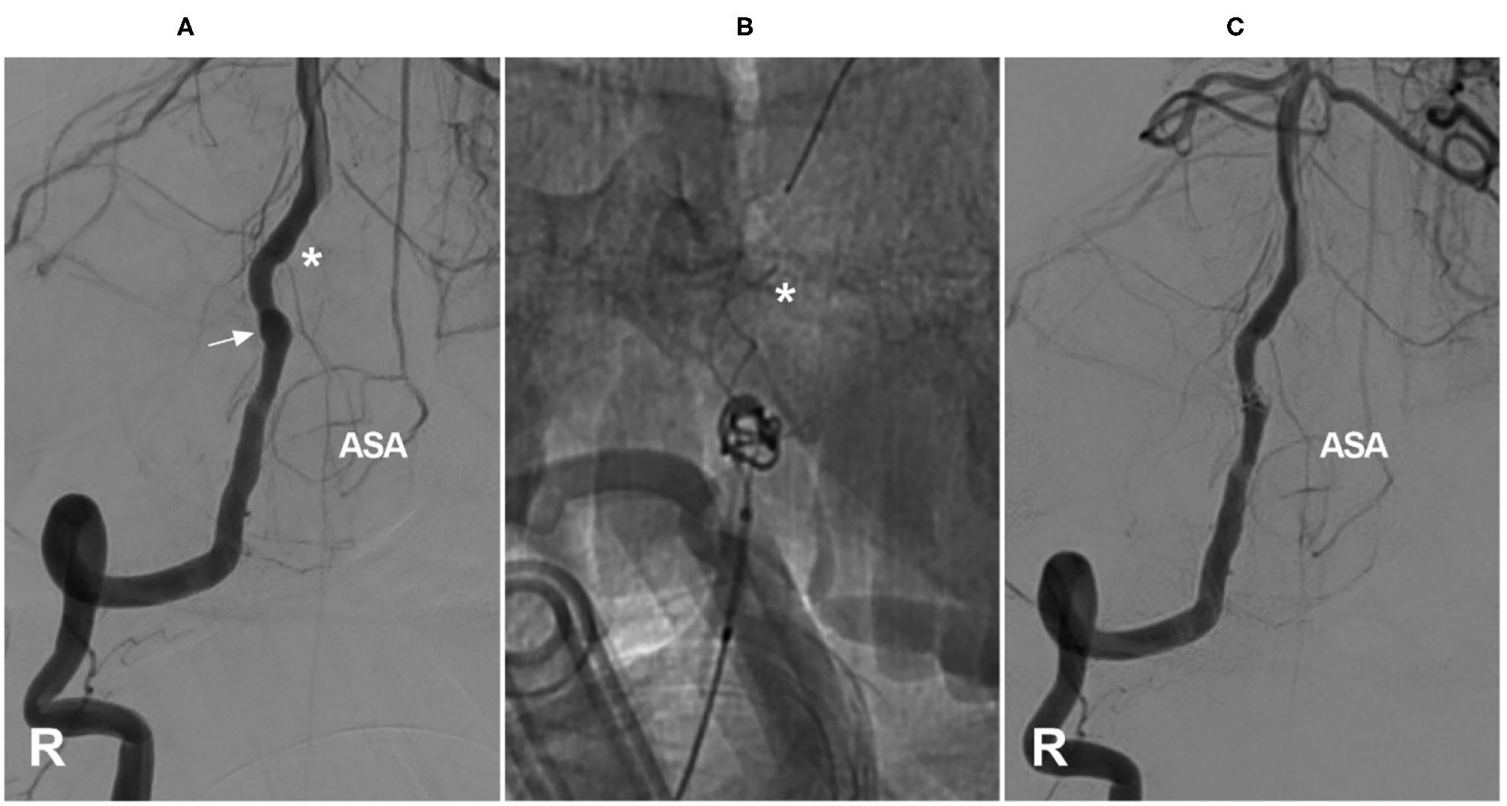
Coverage of the ASA ostia after coiling with stent assistance for an aneurysm. **(A)** Right VA angiogram showing the dissecting aneurysm (arrow) and the ASA (the asterisk indicates its origin). **(B)** The aneurysm was coiled with Leo stent assistance, and the tip of the stent covered the ASA ostia (asterisk). **(C)** After coiling, the ASA had normal blood flow. ASA, anterior spinal artery; R, right; VA, vertebral artery.

The metal coverage of conventional intracranial stents is low; with their assistance, coiling for VA aneurysms is safe for the ASA (Figure 5) (97). However, when the ASA originates from the aneurysm, coiling with sacrifice of the ASA should be conducted with caution (11). If the collateral circulation is insufficient, occlusion of an unpaired ASA can result in bilateral medial medullary syndrome (11, 98, 99).

Is VA trapping safe? In an Aihara et al., study on the predictive factors of medullary infarction after VA trapping for aneurysms, 30% of the patients suffered medullary infarction. The study showed that the risk was not the length but the anatomical location of VA trapping; therefore, preservation of the ASA origin can reduce the risk of medullary infarction (100). In addition, it is worth noting that, sometimes, despite preserved flow of the ASA, spinal cord hemodynamic infarction can occur due to hypoperfusion (11).

### FD Deployment

The VA has fewer critical perforators, and FD deployment in the VA is always considered a safe choice. However, in theory, because the occlusion of an unpaired ASA can result in medial medullary syndrome, FD deployment in the VA still causes concern regarding medullary ischemia from ASA ostia coverage (101, 102).

What is the consequence in the real world? In a recent multicenter study by Adam et al., VA aneurysms were treated by FD, ASAs were identified in 80.9% of aneurysms, 55.6% of ASAs were covered by the FD, and patency after FD coverage at the last follow-up was 89.2% for ASAs. The study showed that FD deployment with coverage of the ASA was not associated with higher rates of occlusion of the ASA or any instances of cord infarction (7).

**Figure 6:**
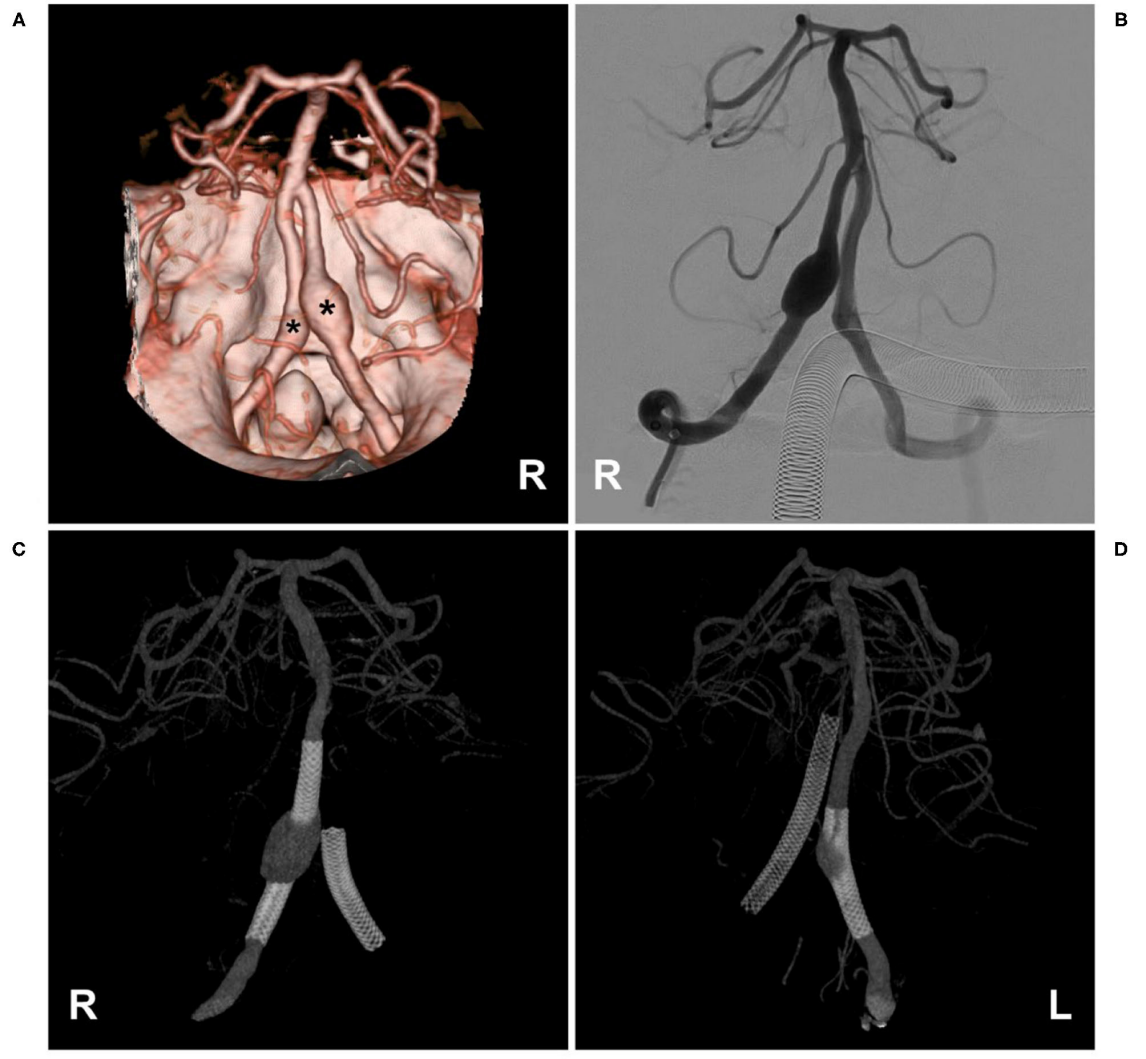
Flow diversion deployment to treat bilateral VA aneurysms. **(A,B)** CTA **(A)** and VA angiogram **(B)** showing bilateral VA dissecting aneurysms (asterisks in **(A)**); in **(B)**, the ASA was not observed. **(C,D)** Angiogram of the bilateral VA showing the flow diversions deployed to treat the aneurysms. ASA, anterior spinal artery; CTA, computed tomography angiography; L, left; R, right; VA, vertebral artery.

Considering the potential risk factor, FD deployment should likely be avoided in cases of an unpaired ASA, and FD with coverage of the sole ASA is likely not a favorable treatment option (11). In cases of paired ASAs, FD use could be more liberal; however, FD deployment in bilateral VAs necessitates more caution (Figure 6).

### VA Stent Angioplasty and Balloon Angioplasty

**Figure 7:**
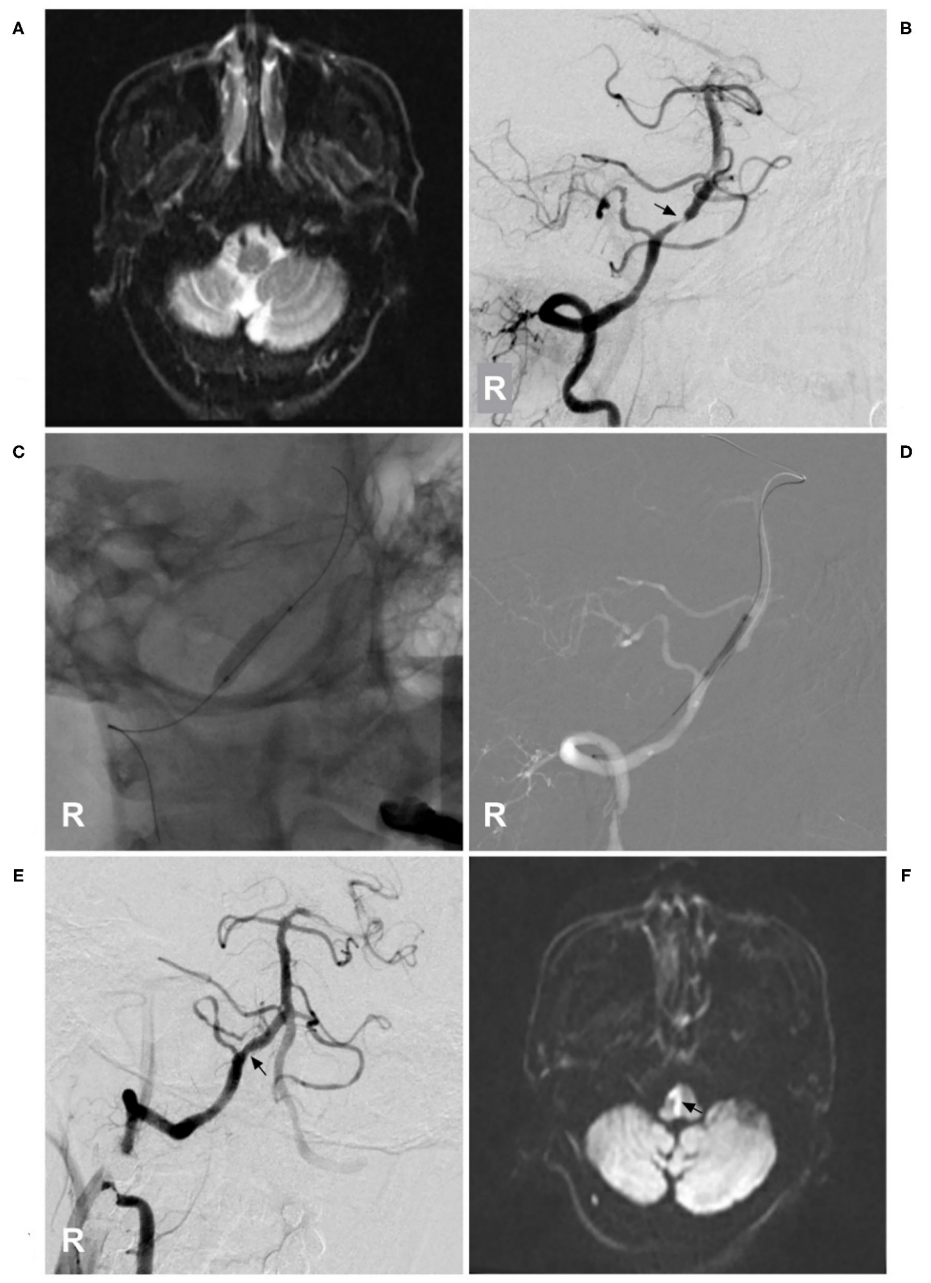
Medulla oblongata infarction from ASA ischemia after stent angioplasty. **(A)** MRI showed a normal medulla oblongata. **(B)** A right VA angiogram showed stenosis (arrow) of the VA beyond the posterior inferior cerebellar artery. **(C)** Balloon angioplasty was performed. **(D)** A balloon-expandable stent was deployed. **(E)** After stent angioplasty, the stenosis was relieved (arrow). **(F)** Postoperative MRI showed acute medulla oblongata infarction (arrow), indicating ASA ischemia and resulting in locked-in syndrome. ASA, anterior spinal artery; MRI, magnetic resonance imaging; R, right; VA, vertebral artery.

Acute VA dissection can result in occlusion of the ASA (94, 103). Therefore, there is a danger that intracranial VA stent angioplasty or balloon angioplasty of the VA may injure the ASA ostia, where the dissection formed. In the study of Wang et al. of 55 cases with intracranial VA stenosis, balloon angioplasty and stent angioplasty resulted in 9.1% of cases with perforator injuries of the VA, resulting in ischemic complications, most of which resulted from ASA injury (104). Therefore, the ASA should be identified. If the ASA is not seen, dissection of its region of origin should be avoided (Figure 7).

### Injury From a Supporting Catheter

**Figure 8:**
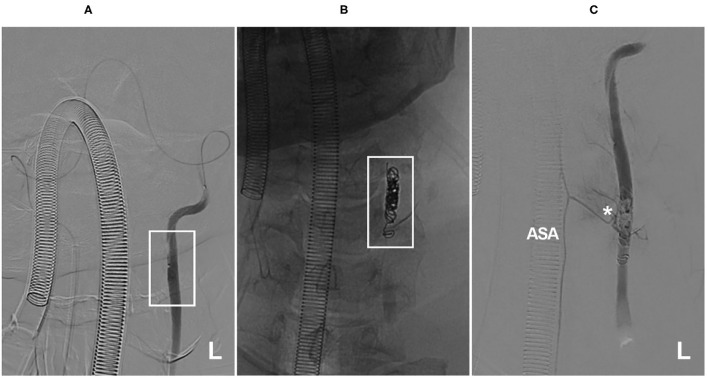
Insufficiency of the artery of cervical enlargement after VA occlusion. **(A)** A microcatheter angiogram showed that the microcatheter reached the left VA (frame) from the right to perform VA occlusion, and no segmental artery was observed. **(B)** X-ray film showed left VA occlusion by coiling (frame). **(C)** A microcatheter angiogram showed that the coils occluded the origin of the artery of cervical enlargement (asterisk). ASA, anterior spinal artery; L, left; VA, vertebral artery. The patient was a 56-year-old man. He suffered neck bleeding from the VA; thus, the VA was occluded. After the treatment, he had no new neurological deficits.

In general, the occlusion of an artery feeding the cervical regions rarely results in an infarction of the spinal cord, as these areas have well-vascularized networks (105). However, similar to occlusion of the artery of Adamkiewicz, which can result in spinal cord infarction, insufficiency of the artery of cervical enlargement is a dangerous situation (Figure 8) (106, 107).

During EVT *via* the VA, a larger-sized guiding catheter is often necessary; it can restrict blood flow and produce catheter-induced vasospasm, which can rarely result in thromboemboli and/or hemodynamic insufficiency of the artery of cervical enlargement, resulting in ASA ischemia (24). Therefore, excise VA angiography is recommended, and the catheter should be placed away from the ostia of the artery of cervical enlargement. The guiding catheter should also be continuously flushed with heparinized saline (108).

## Cervical ASA as a Collateral Channel

**Figure 9:**
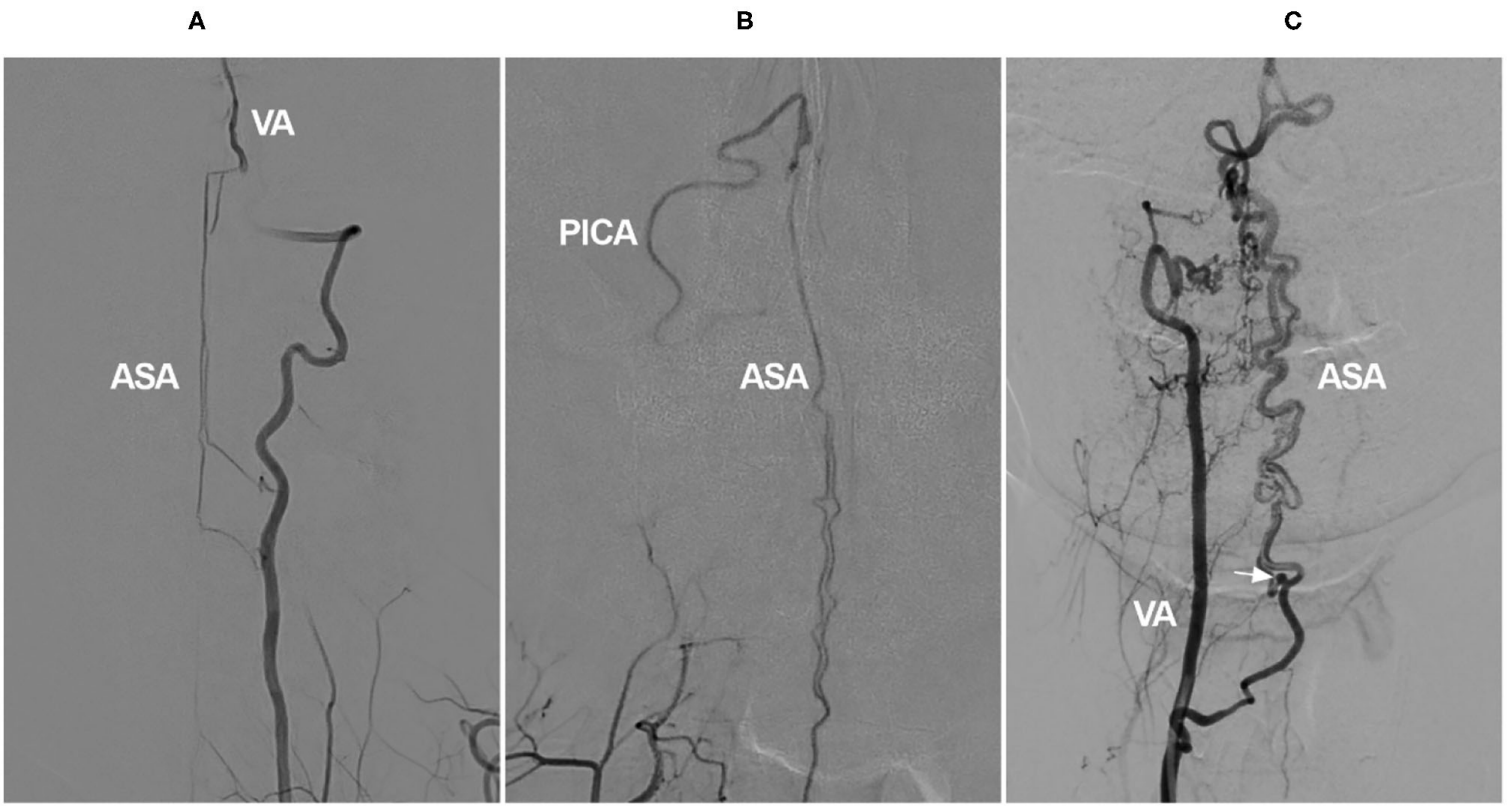
Cervical ASA as a collateral channel in VA occlusion. **(A)** Angiogram of the VA shows that the intracranial VA is not continuous, and the ASA connects the proximal and distal segments of VA. **(B)** Angiogram of the VA shows that there are double branches of ASA, and the ASA connects to the PICA. **(C)** Angiogram of the VA shows the VA rete mirabile; the ASA is tortuous and dilated to provide blood to the posterior circulation, and an aneurysm is found (arrow). ASA, anterior spinal artery; PICA, posterior inferior cerebellar artery; VA, vertebral artery.

Proximal VA occlusion at the neck is usually compensated *via* the thyrocervical, deep cervical, occipital, and ascending pharyngeal arteries (109). Rarely, retrograde flow through the ASA can serve as a collateral channel, especially in chronic bilateral VA occlusion or a single VA occlusion with other severe stenoses (Figures 9A,B) (5). In addition, in VA rete mirabile, the ASA may also serve as the collateral channel (Figure 9C) (110).

The degree of the ASA collateral channel varies depending on the presence of other collateral routes (109). Due to the small diameter of the ASA, collateral flow through the ASA may be unable to compensate for critical hypoperfusion, resulting in recurrent strokes (111). VA stenting can markedly improve the flow of the posterior circulation; after successful recanalization of the occluded VA, the collateral channel of the ASA may disappear (5, 112).

**Figure 10:**
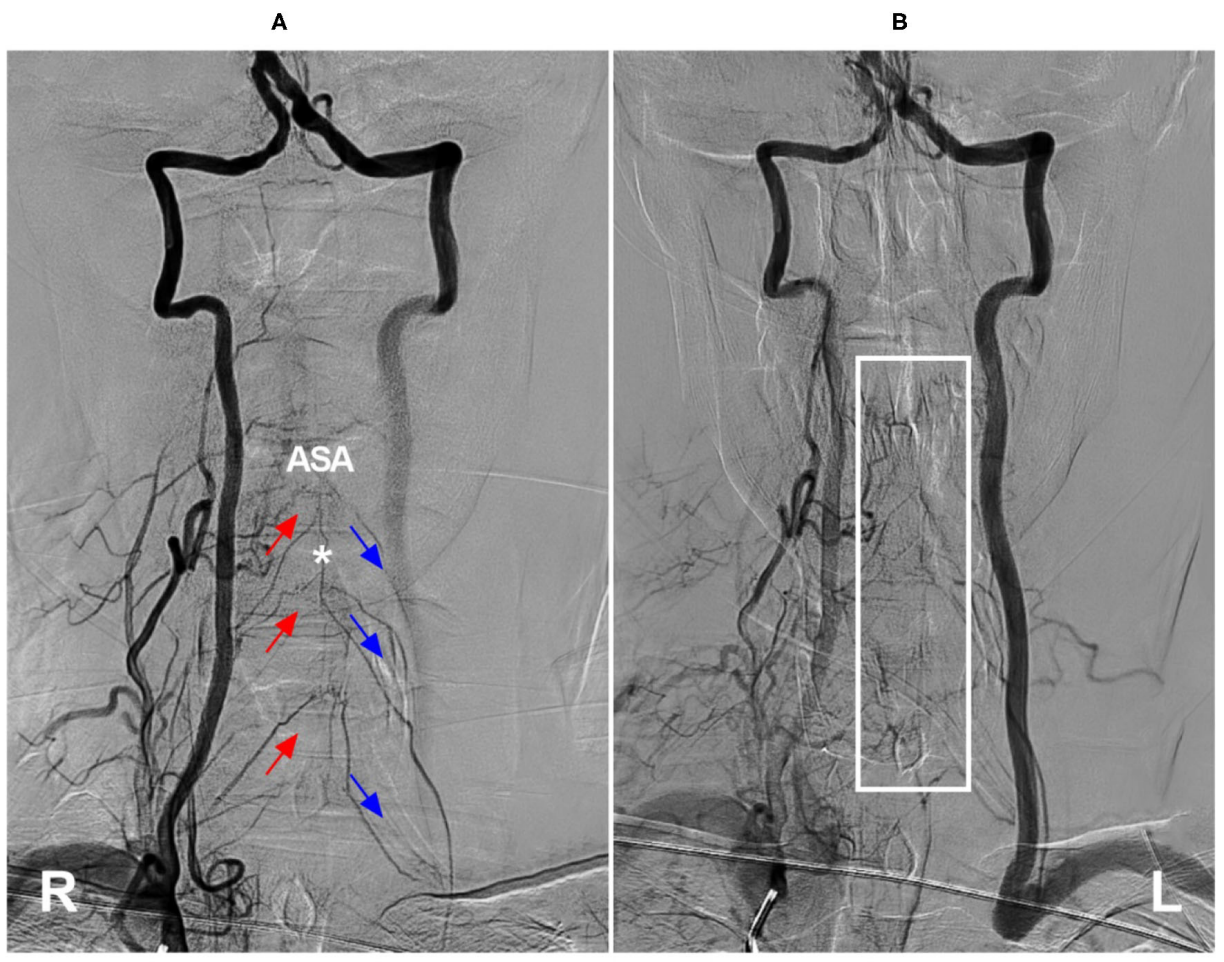
Cervical ASA as a collateral channel in steno-occlusive disease of the subclavian artery. **(A)** Angiogram of the right VA in arterial phase shows that the compensation of collateral circulation from the right segmental radiculomedullary arteries (red arrows) to the left radiculomedullary arteries (blue arrows) through ASA (asterisk). **(B)** Angiogram of the right VA in late arterial phase shows the capillary dyeing sign in ASA region (frame). ASA, anterior spinal artery; L, left; R, right; VA, vertebral artery.

Other than in cases of VA occlusion and rete mirabile, the ASA as a collateral channel can occur in the subclavian steal phenomenon, in which the artery of cervical enlargement may stem from the normal VA to connect to a spinal branch of the contralateral VA or costocervical trunk, resulting in ASA syndrome presenting as cervical myelopathy (Figure 10) (113, 114). At this time, the subclavian artery should be reconstructed, and the steal path should be occluded (114).

**Figure 11:**
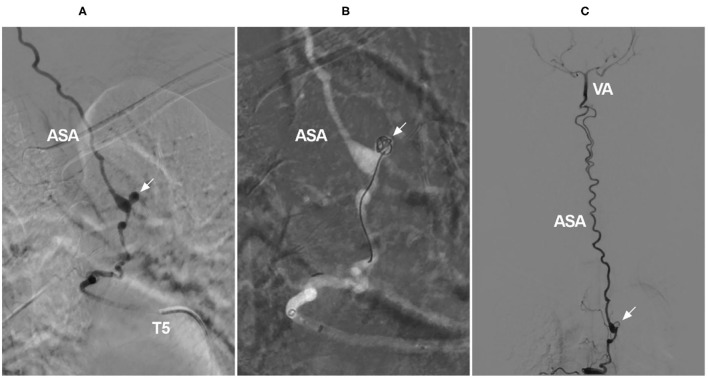
Coiling of a ruptured aneurysm on the ASA as a collateral channel. **(A)** Angiogram of the T5 intercostal artery showing an aneurysm on the ASA (arrow), the ASA served as an upward collateral channel. **(B)** Under the roadmap, the aneurysm was coiled (arrow). **(C)** Overview of the angiogram showing that the aneurysm was coiled (arrow) and that the ASA was connected to the intracranial VA. ASA, anterior spinal artery; T, thoracic; VA, vertebral artery.

When the ASA acts as a collateral channel, aneurysms can occur on the ASA due to hemodynamic stress (Figure 9C) (115). ASA aneurysms can be coiled in selected cases (Figure 11) (116, 117). However, due to the tortuous path and remote location, coiling is often difficult and impossible because EVT has a low likelihood of parent artery preservation with the latter option (118).

## Summary

The cervical ASA is a very important artery. It can be involved in many cervical vascular diseases and has many different roles. In AVF and AVM, the cervical ASA often acts as an accomplice or victim because it acts as the feeder. In ASA aneurysm, the cervical ASA is a victim. During EVT for VA diseases, the cervical ASA ostia can be covered or occluded. In such cases, the ASA is a victim. In VA occlusion, the cervical ASA can serve as a collateral channel to provide blood flow to the posterior circulation. In this situation, the cervical ASA plays the role of a friend. In summary, EVT for cervical spinal vascular diseases should be determined “case by case,” and damage to the ASA axis should be avoided.

## Author Contributions

JY contributed to the conception, design of the manuscript, and critically revised the manuscript. KZ and CL wrote the manuscript. KH and CL collected the medical records of the patients. All authors approved the final version of this manuscript.

## Conflict of Interest

The authors declare that the research was conducted in the absence of any commercial or financial relationships that could be construed as a potential conflict of interest.

## Publisher's Note

All claims expressed in this article are solely those of the authors and do not necessarily represent those of their affiliated organizations, or those of the publisher, the editors and the reviewers. Any product that may be evaluated in this article, or claim that may be made by its manufacturer, is not guaranteed or endorsed by the publisher.

## References

[1] Ali F Reddy V Dublin AB. Anatomy, Back, Anterior Spinal Artery. Treasure Island, FL: StatPearls (2021). 30422558

[2] Mizutani K Consoli A Maria FD Condette Auliac S Boulin A Coskun O. Intradural spinal cord arteriovenous shunts in a personal series of 210 patients: novel classification with emphasis on anatomical disposition and angioarchitectonic distribution, related to spinal cord histogenetic units. J Neurosurg Spine. (2021) 34: 920–30. 10.3171/2020.9.SPINE201258 33799293

[3] Yamazaki D Hanaoka Y Koyama JI Suzuki Y Agata M Abe D. Intraspinal canal platform system for coil embolization of anterior spinal artery aneurysm associated with spinal cord arteriovenous malformation: a case report and literature review. Br J Neurosurg. (2021) 1–6. 10.1080/02688697.2021.1910201 33851560

[4] Abdalkader M Samuelsen BT Moore JM Cervantes-Arslanian A Ong CJ Setty BN. Ruptured spinal aneurysms: diagnosis and management paradigms. World Neurosurg. (2021) 146:e368–77. 10.1016/j.wneu.2020.10.098 33223127

[5] Kang HS Han MH Kim SH Kwon OK Roh HG Koh YC. Anterior spinal artery as a collateral channel in cases of bilateral vertebral arterial steno-occlusive diseases. AJNR Am J Neuroradiol. (2007) 28:222–5. 17296984 PMC7977389

[6] Hiramatsu M Sugiu K Ishiguro T Kiyosue H Sato K Takai K. Angioarchitecture of arteriovenous fistulas at the craniocervical junction: a multicenter cohort study of 54 patients. J Neurosurg. (2018) 128:1839–49. 10.3171/2017.3.JNS163048 28862546

[7] Dmytriw AA Kapadia A Enriquez-Marulanda A Parra-Farinas C Kuhn AL Nicholson PJ. Vertebral artery aneurysms and the risk of cord infarction following spinal artery coverage during flow diversion. J Neurosurg. (2020) 134:961–70. 10.3171/2020.1.JNS193293 32217800

[8] Yu JX He C Ye M Li GL Bian LS Yang F. The efficacy and deficiency of contemporary treatment for spinal cord arteriovenous shunts. Brain. (2021). 10.1093/brain/awab237 34156437

[9] Rojas S Ortega M Rodriguez-Baeza A. Anatomical study of the pial arterial network in human spinal cords. Clin Anat. (2021) 34:596–604. 10.1002/ca.23622 32427384

[10] Arslan M Acar HI Comert A Tubbs RS. The cervical arteries: an anatomical study with application to avoid the nerve root and spinal cord blood supply. Turk Neurosurg. (2018) 28:234–40. 10.5137/1019-5149.JTN.19469-16.1 28266004

[11] Ravina K Strickland BA Rennert RC Fredrickson V Bakhsheshian J Chien M. Fusiform vertebral artery aneurysms involving the posterior inferior cerebellar artery origin associated with the sole angiographic anterior spinal artery origin: technical case report and treatment paradigm proposal. J Neurosurg. (2018) 131: 1324–30. 10.3171/2018.5.JNS18681 30485231

[12] Rodriguez-Baeza A Muset-Lara A Rodriguez-Pazos M Domenech-Mateu JM. Anterior spinal arteries. Origin and distribution in man. Acta Anat. (1989) 136:217–21. 10.1159/000146889 2603634

[13] Santos-Franco JA de Oliveira E Mercado R Ortiz-Velazquez RI Revuelta-Gutierrez R Gomez-Llata S. Microsurgical considerations of the anterior spinal and the anterior-ventral spinal arteries. Acta Neurochir. (2006) 148:329–38, discussion 38. 10.1007/s00701-005-0663-7 16328774

[14] Sheehy NP Boyle GE Meaney JF. Normal anterior spinal arteries within the cervical region: high-spatial-resolution contrast-enhanced three-dimensional MR angiography. Radiology. (2005) 236:637–41. 10.1148/radiol.2362040804 15972334

[15] Kanazawa J Yan J Hitomi J. Differences in distribution of anterior segmental medullary arteries in the cervical and thoracolumbar spinal cord: the “inseln” were characteristics in the cervical spinal cord. Anat Sci Int. (2020) 95:97–103. 10.1007/s12565-019-00498-y 31399898 PMC6942566

[16] Dunbar L Vidakovic H Loffler S Hammer N Gille O Boissiere L. Anterior cervical spine blood supply: a cadaveric study. Surg Radiol Anat. (2019) 41:607–11. 10.1007/s00276-019-02236-5 30937565

[17] Oh SH Perin NI Cooper PR. Quantitative three-dimensional anatomy of the subaxial cervical spine: implication for anterior spinal surgery. Neurosurgery. (1996) 38:1139–44. 10.1227/00006123-199606000-00016 8727144

[18] Inci S Bertan V Cila A. Angiographically occult epidural arteriovenous fistula of the craniocervical junction. Surg Neurol. (2002) 57:167–73, discussion 73. 10.1016/S0090-3019(02)00631-6 12009541

[19] Lasjaunias P Vallee B Person H Ter Brugge K Chiu M. The lateral spinal artery of the upper cervical spinal cord. Anatomy, normal variations, and angiographic aspects. J Neurosurg. (1985) 63:235–41. 10.3171/jns.1985.63.2.0235 4020445

[20] Gailloud P. The supreme intercostal artery includes the last cervical intersegmental artery (C7) - angiographic validation of the intersegmental nomenclature proposed by Dorcas Padget in 1954. Anat Rec. (2014) 297:810–8. 10.1002/ar.22893 24610867

[21] Boeris D Mortimer A Sakthithasan M Evins AI Sandeman D Renowden S. Conservative management of a post-traumatic pseudoaneurysm of the artery of cervical enlargement-anterior spinal artery junction. BMJ Case Rep. (2015) 1–3. 10.1136/bcr-2015-011662 25809436 PMC4386298

[22] Sherman PM Gailloud P. Artery of the cervical enlargement originating from the inferior thyroid artery: an angiographic observation. J Vasc Interv Radiol. (2004) 15:648–50. 10.1016/S1051-0443(07)60341-8 15178730

[23] Miller DL. Direct origin of the artery of the cervical enlargement from the left subclavian artery. AJNR Am J Neuroradiol. (1993) 14:242–4. 8427098 PMC8334463

[24] Matsubara N Miyachi S Okamaoto T Izumi T Asai T Yamanouchi T. Spinal cord infarction is an unusual complication of intracranial neuroendovascular intervention. Interv Neuroradiol. (2013) 19:500–5. 10.1177/159101991301900416 24355157 PMC3902752

[25] Brinjikji W Colombo E Lanzino G. Clinical and angioarchitectural characteristics of spinal vascular malformations of the cervical spine. J Neurosurg Spine. (2020) 32:755–62. 10.3171/2019.11.SPINE19798 31952038

[26] Dabus G Nimmagadda A Russell EJ. Cervical epidural arteriovenous fistula presenting with radiculopathy: transvenous embolization using Onyx. Interv Neuroradiol. (2011) 17:380–5. 10.1177/159101991101700317 22005704 PMC3396030

[27] Fujimoto S Takai K Nakatomi H Kin T Saito N. Three-dimensional angioarchitecture and microsurgical treatment of arteriovenous fistulas at the craniocervical junction. J Clin Neurosci. (2018) 53:140–6. 10.1016/j.jocn.2018.04.065 29731281

[28] Takai K. Spinal arteriovenous shunts: angioarchitecture and historical changes in classification. Neurol Med Chir. (2017) 57:356–65. 10.2176/nmc.ra.2016-0316 28515372 PMC5566708

[29] Noh T Chandra R Kim J Lee I. A case of symptomatic spinal dural arteriovenous fistula after high-volume lumbar puncture. Surg Neurol Int. (2017) 8:164. 10.4103/sni.sni_474_16 28840068 PMC5551290

[30] Krings T Geibprasert S. Spinal dural arteriovenous fistulas. AJNR Am J Neuroradiol. (2009) 30:639–48. 10.3174/ajnr.A1485 19213818 PMC7051782

[31] Park SB Han MH Jahng TA Kwon BJ Chung CK. Spinal dural arteriovenous fistulas: clinical experience with endovascular treatment as a primary therapeutic modality. J Korean Neurosurg Soc. (2008) 44:364–9. 10.3340/jkns.2008.44.6.364 19137080 PMC2615139

[32] Koch MJ Stapleton CJ Agarwalla PK Torok C Shin JH Coumans JV. Patel Open and endovascular treatment of spinal dural arteriovenous fistulas: a 10-year experience. J Neurosurg Spine. (2017) 26:519–23. 10.3171/2016.9.SPINE16394 28106525

[33] Safaee MM Clark AJ Burkhardt JK Winkler EA Lawton MT. Timing, severity of deficits, and clinical improvement after surgery for spinal dural arteriovenous fistulas. J Neurosurg Spine. (2018) 29:85–91. 10.3171/2017.11.SPINE17988 29676670

[34] Su H Xu K Wang Y Yu J. Is the middle meningeal artery the optimal path for dural arteriovenous fistula embolization? Front Neurol. (2021) 12:675355. 10.3389/fneur.2021.675355 34135854 PMC8201068

[35] Jeon JP Cho YD Kim CH Han MH. Complex spinal arteriovenous fistula of the craniocervical junction with pial and dural shunts combined with contralateral dural arteriovenous fistula. Interv Neuroradiol. (2015) 21:733–7. 10.1177/1591019915609128 26464289 PMC4757362

[36] Nguyen A Maynard K III Coggins W Raghuram K. Successful embolization of an upper cervical spinal dural fistula despite anterior spinal artery anastomosis. Br J Neurosurg. (2019) 1–3. 10.1080/02688697.2019.1694136 31760851

[37] Adrianto Y Yang KH Koo HW Park W Jung SC Park JE. Concomitant origin of the anterior or posterior spinal artery with the feeder of a spinal dural arteriovenous fistula (SDAVF). J Neurointerv Surg. (2017) 9:405–10. 10.1136/neurintsurg-2016-012267 27060157

[38] Hayward DM Johans SJ Rosenblum JD Loftus CM Ashley WW. Balloon-occlusion catheter onyx embolization of a spinal dural arteriovenous fistula presenting with subarachnoid hemorrhage in a pediatric patient. J Stroke Cerebrovasc Dis. (2016) 25:e46–9. 10.1016/j.jstrokecerebrovasdis.2015.12.040 26851209

[39] Onda K Yoshida Y Watanabe K Arai H Okada H Terada T. High cervical arteriovenous fistulas fed by dural and spinal arteries and draining into a single medullary vein: report of 3 cases. J Neurosurg Spine. (2014) 20:256–64. 10.3171/2013.11.SPINE13402 24438426

[40] Takai K Komori T Kurita H Kawai K Inoue T Taniguchi M. Intradural radicular arteriovenous fistula that mimics dural arteriovenous fistula: report of three cases. Neuroradiology. (2019) 61:1203–8. 10.1007/s00234-019-02275-0 31396663

[41] Mathur S Symons SP Huynh TJ Muthusami P Montanera W Bharatha A. First-pass contrast-enhanced MRA for pretherapeutic diagnosis of spinal epidural arteriovenous fistulas with intradural venous reflux. AJNR Am J Neuroradiol. (2017) 38:195–9. 10.3174/ajnr.A5008 27884880 PMC7963659

[42] Yu JX Hong T Ma YJ Ling F Zhang HQ. A new type of spinal epidural arteriovenous fistulas causes spinal epidural hemorrhage: an analysis of five cases and natural history consideration. World Neurosurg. (2017) 103:371–9. 10.1016/j.wneu.2017.04.036 28427979

[43] Brinjikji W Yin R Nasr DM Lanzino G. Spinal epidural arteriovenous fistulas. J Neurointerv Surg. (2016) 8:1305–10. 10.1136/neurintsurg-2015-012181 26790829

[44] Asai J Hayashi T Fujimoto T Suzuki R. Exclusively epidural arteriovenous fistula in the cervical spine with spinal cord symptoms: case report. Neurosurgery. (2001) 48:1372–5, discussion 5–6. 10.1227/00006123-200106000-00043 11383745

[45] Song Y Cho SH Lee DW Sheen JJ Shin JH Suh DC. Osseous versus nonosseous spinal epidural arteriovenous fistulas: experiences of 13 patients. AJNR Am J Neuroradiol. (2019) 40:129–34. 10.3174/ajnr.A5904 30523143 PMC7048584

[46] Suh DC Choi CG Sung KB Kim KK Rhim SC. Spinal osseous epidural arteriovenous fistula with multiple small arterial feeders converging to a round fistular nidus as a target of venous approach. AJNR Am J Neuroradiol. (2004) 25:69–73. 14729531 PMC7974159

[47] Takai K Shojima M Imai H Saito N Taniguchi M. Microsurgical and endovascular treatments of spinal extradural arteriovenous fistulas with or without intradural venous drainage. World Neurosurg. (2018) 111:e819–29. 10.1016/j.wneu.2017.12.162 29309979

[48] Rangel-Castilla L Holman PJ Krishna C Trask TW Klucznik RP Diaz OM. Spinal extradural arteriovenous fistulas: a clinical and radiological description of different types and their novel treatment with Onyx. J Neurosurg Spine. (2011) 15:541–9. 10.3171/2011.6.SPINE10695 21800954

[49] Takai K Taniguchi M. Comparative analysis of spinal extradural arteriovenous fistulas with or without intradural venous drainage: a systematic literature review. Neurosurg Focus. (2012) 32:E8. 10.3171/2012.2.FOCUS1216 22537134

[50] Nakagawa I Park HS Hironaka Y Wada T Kichikawa K Nakase H. Cervical spinal epidural arteriovenous fistula with coexisting spinal anterior spinal artery aneurysm presenting as subarachnoid hemorrhage–case report. J Stroke Cerebrovasc Dis. (2014) 23:e461–5. 10.1016/j.jstrokecerebrovasdis.2014.07.012 25284720

[51] Yoshida K Sato S Inoue T Ryu B Shima S Mochizuki T. Transvenous embolization for craniocervical junction epidural arteriovenous fistula with a pial feeder aneurysm. Interv Neuroradiol. (2020) 26:170–7. 10.1177/1591019919874571 31488023 PMC7507230

[52] Huang W Gross BA Du R. Spinal extradural arteriovenous fistulas: clinical article. J Neurosurg Spine. (2013) 19:582–90. 10.3171/2013.8.SPINE13186 24033305

[53] Goto Y Hino A Shigeomi Y Oka H. Surgical management for craniocervical junction arteriovenous fistula targeting the intradural feeder. World Neurosurg. (2020) 144:e685–92. 10.1016/j.wneu.2020.09.041 32942059

[54] Elkordy A Endo T Sato K Sonoda Y Takahashi A Tominaga T. Exclusively epidural spinal metameric arteriovenous shunts: case report and literature review. Spine J. (2015) 15:e15–22. 10.1016/j.spinee.2014.11.022 25450654

[55] Shetty GS Singh V Prasad SN Phadke RV Neyaz Z Udiya A. Spinal epidural fistulas-a separate entity to dural fistulas with different angioarchitecture and treatment approach. World Neurosurg. (2021) 149:e600–e11. 10.1016/j.wneu.2021.01.126 33548535

[56] Umana GE Scalia G Chaurasia B Fricia M Passanisi M Graziano F. Perimedullary arteriovenous fistulas of the craniovertebral junction: a systematic review. J Craniovertebr Junction Spine. (2020) 11:157–62. 10.4103/jcvjs.JCVJS_106_20 33100763 PMC7546045

[57] Ji T Guo Y Shi L Yu J. Study and therapeutic progress on spinal cord perimedullary arteriovenous fistulas. Biomed Rep. (2017) 7:214–20. 10.3892/br.2017.951 28808569 PMC5543424

[58] Endo T Shimizu H Sato K Niizuma K Kondo R Matsumoto Y. Cervical perimedullary arteriovenous shunts: a study of 22 consecutive cases with a focus on angioarchitecture and surgical approaches. Neurosurgery. (2014) 75:238–49, discussion 49. 10.1227/NEU.0000000000000401 24867200

[59] Spetzler RF Detwiler PW Riina HA Porter RW. Modified classification of spinal cord vascular lesions. J Neurosurg. (2002) 96:145–56. 10.3171/spi.2002.96.2.0145 12450276

[60] Kim LJ Spetzler RF. Classification and surgical management of spinal arteriovenous lesions: arteriovenous fistulae and arteriovenous malformations. Neurosurgery. (2006) 59:S195–201, discussion S3–13. 10.1227/01.NEU.0000237335.82234.CE 17053603

[61] Cho WS Kim KJ Kwon OK Kim CH Kim J Han MH. Clinical features and treatment outcomes of the spinal arteriovenous fistulas and malformation: clinical article. J Neurosurg Spine. (2013) 19:207–16. 10.3171/2013.4.SPINE12732 23705629

[62] Roccatagliata L Kominami S Krajina A Sellar R Soderman M van den Berg R. Spinal cord arteriovenous shunts of the ventral (anterior) sulcus: anatomical, clinical, and therapeutic considerations. Neuroradiology. (2017) 59:289–96. 10.1007/s00234-017-1789-z 28251329

[63] Meng X Zhang H Wang Y Ye M He C Du J. Perimedullary arteriovenous fistulas in pediatric patients: clinical, angiographical, and therapeutic experiences in a series of 19 cases. Childs Nerv Syst. (2010) 26:889–96. 10.1007/s00381-009-1071-8 20107995

[64] Casasco A Guimaraens L Senturk C Cotroneo E Gigli R Theron J. Endovascular treatment of cervical giant perimedullary arteriovenous fistulas. Neurosurgery. (2012) 70:141–9, discussion 9. 10.1227/NEU.0b013e31822ec19e 21796011

[65] Ioannidis I Sfakianos G Nasis N Prodromou P Andreou A. Successful embolization of a giant perimedullary arteriovenous fistula of the cervical spine in a 6-year-old child. Childs Nerv Syst. (2007) 23:1327–30. 10.1007/s00381-007-0385-7 17551737

[66] Zozulya YP Slin'ko EI Al-Qashqish II. Spinal arteriovenous malformations: new classification and surgical treatment. Neurosurg Focus. (2006) 20:E7. 10.3171/foc.2006.20.5.8 16711664

[67] Rangel-Castilla L Russin JJ Zaidi HA Martinez-Del-Campo E Park MS Albuquerque FC. Contemporary management of spinal AVFs and AVMs: lessons learned from 110 cases. Neurosurg Focus. (2014) 37:E14. 10.3171/2014.7.FOCUS14236 25175433

[68] Ohata K Takami T El-Naggar A Morino M Nishio A Inoue Y. Posterior approach for cervical intramedullary arteriovenous malformation with diffuse-type nidus. Report of three cases. J Neurosurg. (1999) 91:105–11. 10.3171/spi.1999.91.1.0105 10419354

[69] Velat GJ Chang SW Abla AA Albuquerque FC McDougall CG Spetzler RF. Microsurgical management of glomus spinal arteriovenous malformations: pial resection technique: clinical article. J Neurosurg Spine. (2012) 16:523–31. 10.3171/2012.3.SPINE11982 22482421

[70] Ducruet AF Crowley RW McDougall CG Albuquerque FC. Endovascular management of spinal arteriovenous malformations. J Neurointerv Surg. (2013) 5:605–11. 10.1136/neurintsurg-2012-010487 22935350

[71] Narayanan S Hurst RW Abruzzo TA Albuquerque FC Blackham KA Bulsara KR. Standard of practice: embolization of spinal arteriovenous fistulae, spinal arteriovenous malformations, and tumors of the spinal axis. J Neurointerv Surg. (2013) 5:3–5. 10.1136/neurintsurg-2012-010551 23112254

[72] Corkill RA Mitsos AP Molyneux AJ. Embolization of spinal intramedullary arteriovenous malformations using the liquid embolic agent, onyx: a single-center experience in a series of 17 patients. J Neurosurg Spine. (2007) 7:478–85. 10.3171/SPI-07/11/478 17977188

[73] Kitamura G Jacobson JP Zouros A Neglio H. Improved neurological function in a paediatric patient following Onyx embolization of a cervical glomus arteriovenous malformation. J Neurointerv Surg. (2010) 2:394–8. 10.1136/jnis.2009.001453 21990655

[74] Flores BC Klinger DR White JA Batjer HH. Spinal vascular malformations: treatment strategies and outcome. Neurosurg Rev. (2017) 40:15–28. 10.1007/s10143-016-0713-z 27075861

[75] Rodesch G Hurth M Alvarez H Lasjaunias P. Embolisation of spinal cord arteriovenous malformations with glue through the anterior spinal axis. Review of 20 Cases. Interv Neuroradiol. (1997) 3:131–43. 10.1177/159101999700300205 20678351

[76] Acewicz A Richter P Tykocki T Czepiel W Ryglewicz D Dowzenko A. Endovascular treatment of cervical intramedullary arteriovenous malformation. Neurol Neurochir Pol. (2014) 48:223–7. 10.1016/j.pjnns.2014.04.002 24981189

[77] Kim J Lee JB Cho TH Hur JW. Incidental occlusion of anterior spinal artery due to onyx reflux in embolization of spinal type II arteriovenous malformation. Eur Spine J. (2017) 26:75–9. 10.1007/s00586-016-4767-y 27671278

[78] Ye ZP Yang XY Li WS Hou B Guo Y. Microsurgical resection of cervical spinal cord arteriovenous malformations: report of 6 cases. World Neurosurg. (2016) 96:362–9. 10.1016/j.wneu.2016.09.022 27641254

[79] Jung SC Song Y Cho SH Kim J Noh SY Lee SH. Endovascular management of aneurysms associated with spinal arteriovenous malformations. J Neurointerv Surg. (2018) 10:198–203. 10.1136/neurintsurg-2017-013150 28637821

[80] Madhugiri VS Ambekar S Roopesh Kumar VR Sasidharan GM Nanda A. Spinal aneurysms: clinicoradiological features and management paradigms. J Neurosurg Spine. (2013) 19:34–48. 10.3171/2013.3.SPINE121026 23621642

[81] Karakama J Nakagawa K Maehara T Ohno K. Subarachnoid hemorrhage caused by a ruptured anterior spinal artery aneurysm. Neurol Med Chir. (2010) 50:1015–9. 10.2176/nmc.50.1015 21123989

[82] Pahl FH de Oliveira MF Rotta MA Dias GM Rezende AL Rotta JM. Spontaneous resolution of an isolated cervical anterior spinal artery aneurysm after subarachnoid hemorrhage. Surg Neurol Int. (2014) 5:139. 10.4103/2152-7806.141776 25317354 PMC4192925

[83] Baldassarre B Balestrino A D'Andrea A Anania P Ceraudo M Truffelli M. Management of spinal aneurysms associated with arteriovenous malformations: systematic literature review and illustrative case. Eur Spine J. (2021). 10.1007/s00586-021-06881-6 34043050

[84] Kurokawa Y Ikawa F Hamasaki O Hidaka T Yonezawa U Komiyama M. A case of cervical spinal dural arteriovenous fistula with extradural drainage presenting with subarachnoid hemorrhage due to a ruptured anterior spinal artery aneurysm. No Shinkei Geka. (2015) 43:803–11. 10.11477/mf.1436203125 26321694

[85] Gross BA Du R. Spinal pial (type IV) arteriovenous fistulae: a systematic pooled analysis of demographics, hemorrhage risk, and treatment results. Neurosurgery. (2013) 73:141–51, discussion 51. 10.1227/01.neu.0000429848.91707.73 23615108

[86] Biondi A Merland JJ Hodes JE Pruvo JP Reizine D. Aneurysms of spinal arteries associated with intramedullary arteriovenous malformations. I. Angiographic and clinical aspects. AJNR Am J Neuroradiol. (1992) 13:913–22. 1590191 PMC8331717

[87] Konan AV Raymond J Roy D. Transarterial embolization of aneurysms associated with spinal cord arteriovenous malformations. Report of four cases. J Neurosurg. (1999) 90:148–54. 10.3171/spi.1999.90.1.0148 10413143

[88] Lavoie P Raymond J Roy D Guilbert F Weill A. Selective treatment of an anterior spinal artery aneurysm with endosaccular coil therapy. Case report. J Neurosurg Spine. (2007) 6:460–4. 10.3171/spi.2007.6.5.460 17542515

[89] Cobb M Griffin A Karikari I Gonzalez LF. Endovascular treatment of ruptured enlarging dissecting anterior spinal artery aneurysm. World Neurosurg. (2020) 139:e658–62. 10.1016/j.wneu.2020.04.100 32339730

[90] Dabus G Tosello RT Pereira BJA Linfante I Piske RL. Dissecting spinal aneurysms: conservative management as a therapeutic option. J Neurointerv Surg. (2018) 10:451–4. 10.1136/neurintsurg-2017-013566 29212861

[91] Boeris D Mortimer A Sakthithasan M Evins AI Sandeman D Renowden S. Conservative management of a post-traumatic pseudoaneurysm of the artery of cervical enlargement-anterior spinal artery junction. J Neurointerv Surg. (2016) 8:e14. 10.1136/neurintsurg-2015-011662.rep 25817516

[92] Simon-Gabriel CP Urbach H Meckel S. Ruptured fusiform aneurysm of the anterior spinal artery: successful treatment with flow diverter stent placed in the feeding vertebral artery. Clin Neuroradiol. (2018) 28:613–6. 10.1007/s00062-018-0684-2 29651585

[93] Wang Y Zhao C Hao X Wang C Wang Z. Endovascular interventional therapy and classification of vertebral artery dissecting aneurysms. Exp Ther Med. (2014) 8:1409–15. 10.3892/etm.2014.1961 25289031 PMC4186359

[94] Wu Y Li W Xie X Jing Z Lu W Huang L. Endovascular treatment with tirofiban during the acute stage of cervical spinal cord infarction due to vertebral artery dissection. J Spinal Cord Med. (2020) 43:130–3. 10.1080/10790268.2017.1422880 29323631 PMC7006665

[95] Pigman EC Shepherd SM. Cervical anterior spinal artery syndrome associated with cardiopulmonary arrest. Am J Emerg Med. (1991) 9:452–4. 10.1016/0735-6757(91)90213-4 1863301

[96] Takahashi S Yamada T Ishii K Saito H Tanji H Kobayashi T. MRI of anterior spinal artery syndrome of the cervical spinal cord. Neuroradiology. (1992) 35:25–9. 10.1007/BF00588273 1289734

[97] Shi L Xu K Sun X Yu J. Therapeutic progress in treating vertebral dissecting aneurysms involving the posterior inferior cerebellar artery. Int J Med Sci. (2016) 13:540–55. 10.7150/ijms.15233 27429591 PMC4946125

[98] Masson C Pruvo JP Meder JF Cordonnier C Touze E De La Sayette V. Spinal cord infarction: clinical and magnetic resonance imaging findings and short term outcome. J Neurol Neurosurg Psychiatry. (2004) 75:1431–5. 10.1136/jnnp.2003.031724 15377691 PMC1738740

[99] Pathak M Kim RC Pribram H. Spinal cord infarction following vertebral angiography: clinical and pathological findings. J Spinal Cord Med. (2000) 23:92–5. 10.1080/10790268.2000.11753514 10914348

[100] Aihara M Naito I Shimizu T Matsumoto M Asakura K Miyamoto N. Predictive factors of medullary infarction after endovascular internal trapping using coils for vertebral artery dissecting aneurysms. J Neurosurg. (2018) 129:107–13. 10.3171/2017.2.JNS162916 28799869

[101] Tsuruta W Yamamoto T Ikeda G Sato M Ito Y Takigawa T. Spinal cord infarction in the region of the posterior spinal artery after embolization for vertebral artery dissection. Oper Neurosurg. (2018) 15:701–10. 10.1093/ons/opy026 29538687

[102] Shakir HJ Rooney PJ Rangel-Castilla L Yashar P Levy EI. Treatment of iatrogenic V2 segment vertebral artery pseudoaneurysm using Pipeline flow-diverting stent. Surg Neurol Int. (2016) 7:104. 10.4103/2152-7806.196235 28168090 PMC5223396

[103] Wang CM Tsai WL Lo YL Chen JY Wong AM. Unilateral right occipital condyle to C2 level spinal cord infarction associated with ipsilateral vertebral artery stenosis and contralateral vertebral artery dissection: a case report. J Spinal Cord Med. (2011) 34:118–21. 10.1179/107902610X12883422813543 21528635 PMC3066486

[104] Wang Z Wang C Li C Shi M Wang S Yang Y. Stenting for symptomatic intracranial vertebrobasilar artery stenosis in northeast of china: a single-center study. Front Neurol. (2020) 11:609286. 10.3389/fneur.2020.609286 33664703 PMC7920948

[105] Turnbull IM Brieg A Hassler O. Blood supply of cervical spinal cord in man. A microangiographic cadaver study. J Neurosurg. (1966) 24:951–65. 10.3171/jns.1966.24.6.0951 5936484

[106] Fukui T Takaki J Okamoto K. Collateral formation from left lateral thoracic artery to the adamkiewicz artery. Aorta. (2020) 8:175–7. 10.1055/s-0040-1721748 33761561 PMC8043810

[107] Chiras J Gaston A Bouhours PG Heraut LA Henry P Bories J. An application of balloon probes. Preoperative electrophysiological test of the tolerance to temporal occlusion of the artery of cervical enlargement. Neurochirurgie. (1983) 29:289–93. 6579392

[108] Sriganesh K Chatterjee N Singha S. Bispectral index monitoring facilitates early detection of catheter-induced vasospasm during neuro-endovascular procedures. Acta Anaesthesiol Scand. (2009) 53:406–7. 10.1111/j.1399-6576.2008.01827.x 19243332

[109] Yamakawa H Yoshimura S Iwama T. Anterior spinal artery as a collateral channel in patients with acute bilateral vertebral artery occlusions. Two case reports. Neurol Med Chir. (2009) 49:354–8. 10.2176/nmc.49.354 19707001

[110] Xu K Yu TC Wang X Yu JL. Bilateral carotid and vertebral rete mirabile associated with intracranial multiple hemorrhages: a case report and literature review. Int J Clin Exp Med. (2016) 9:12153–62.

[111] Fukuda H Hayashi K Handa A Kurosaki Y Lo B Yamagata S. Reflux of anterior spinal artery predicts recurrent posterior circulation stroke in bilateral vertebral artery disease. Stroke. (2015) 46:3263–5. 10.1161/STROKEAHA.115.011246 26419966

[112] Dubow J Riina H Patsalides A. Vertebral artery stenting in a patient with reversed flow in the anterior spinal artery. J Neurointerv Surg. (2012) 4:114–5. 10.1136/jnis.2010.004150 21990442

[113] Mohassel P Wesselingh R Katz Z McArthur J Gailloud P. Anterior spinal artery syndrome presenting as cervical myelopathy in a patient with subclavian steal syndrome. Neurol Clin Pract. (2013) 3:358–60. 10.1212/CPJ.0b013e318296f217 24195022 PMC3787115

[114] Rughani AI Visioni A Hamill RW Tranmer BI. Subclavian artery stenosis causing transient bilateral brachial diplegia: an unusual cause of anterior spinal artery syndrome. J Neurosurg Spine. (2008) 9:191–5. 10.3171/SPI/2008/9/8/191 18764753

[115] Ashour R Filippidis A Patel N. Ruptured anterior spinal artery aneurysm associated with bilateral vertebral artery occlusion treated by surgical clipping. World Neurosurg. (2015) 84:1178.e11–3. 10.1016/j.wneu.2015.06.010 26079782

[116] Glauser G Hurst RW Pukenas BA Sedora-Roman NI Kung D Choudhri O. Subarachnoid hemorrhage as result of retrocorporeal artery aneurysm rupture: rare sequel of subclavian steal syndrome. World Neurosurg. (2020) 133:66–8. 10.1016/j.wneu.2019.09.129 31574332

[117] Shima S Sato S Motizuki T Niimi Y. Endovascular treatment for ruptured cervical anterior spinal artery aneurysm caused by occlusive disease of bilateral vertebral arteries: a case report and literature review. Clin Neurol Neurosurg. (2021) 208:106862. 10.1016/j.clineuro.2021.106862 34391976

[118] Nagahata M Kondo R Mouri W Sato A Ito M Sato S. Bilateral carotid and vertebral rete mirabile presenting with subarachnoid hemorrhage caused by the rupture of spinal artery aneurysm. Tohoku J Exp Med. (2013) 230:205–9. 10.1620/tjem.230.205 23903351

